# Olive Oil Based Methotrexate Loaded Topical Nanoemulsion Gel for the Treatment of Imiquimod Induced Psoriasis-like Skin Inflammation in an Animal Model

**DOI:** 10.3390/biology10111121

**Published:** 2021-10-31

**Authors:** Sheikh Abdur Rashid, Sajid Bashir, Faiza Naseem, Arshad Farid, Irfan A. Rather, Khalid Rehman Hakeem

**Affiliations:** 1 Gomal Centre of Pharmaceutical Sciences, Faculty of Pharmacy, Gomal University, Dera Ismail Khan 29050, Pakistan; faizanaseem17@gmail.com; 2 College of Pharmacy, University of Sargodha, Sargodha 40100, Pakistan; sajidpharm@gmail.com; 3 Gomal Centre of Biochemistry & Biotechnology, Gomal University, Dera Ismail Khan 29050, Pakistan; arshadfarid@gu.edu.pk; 4 Department of Biological Sciences, Faculty of Science, King Abdulaziz University, Jeddah 21589, Saudi Arabia; 5 Princess Dr. Najla Bint Saud Al- Saud Center for Excellence Research in Biotechnology, King Abdulaziz University, Jeddah 21589, Saudi Arabia

**Keywords:** psoriasis, inflammation, methotrexate, topical delivery, skin permeation

## Abstract

**Simple Summary:**

Psoriasis, being chronic inflammatory illness, provoked by genetic and environmental factors is linked to several other life-threatening diseases. Methotrexate is regarded as gold standard for the management of psoriasis, so an attempt was made to incorporate this drug into nanoemulsion gel. Thus olive oil based formulation was fabricated to target animal model induced psoriasis- like skin inflammation. The optimized methotrexate nanoemulsion gel formulation produced a psoriasis area and severity Index (PASI) decrease that was similar or better than the 91% reduction seen in the methotrexate tablet group. The results of this study revealed effectiveness of methotrexate nanoemulsion gel formulation to treat psoriasis and reduce the remission of psoriasis-like symptoms.

**Abstract:**

Psoriasis, a chronic inflammatory illness, is on the rise and is linked to several other life-threatening diseases. The primary goal of this study was to create a nanoemulsion gel loaded with methotrexate and olive oil (MTX NEG). The formulation was evaluated for physicochemical characterization, entrapment efficiency, drug release kinetics, skin permeation studies and stability tests. In addition, the efficacy of MTX NEG against psoriasis was tested using imiquimod-induced psoriasis in a rat model. The final optimized MTX NEG was developed with a particle size of 202.6 ± 11.59 nm and a PDI of 0.233 ± 0.01, with a 76.57 ± 2.48% average entrapment efficiency. After 20 h, the release kinetics predicted a 72.47% drug release at pH 5.5. FTIR findings demonstrated that the optimized MTX NEG formulation effectively fluidized both the epidermis and dermis of the skin, potentially increasing drug permeability and retention. The application of Tween 80 and PEG 400, on the other hand, significantly enhanced these effects, as these are well known penetration enhancers. After 24 h, an average of 70.78 ± 5.8 μg/cm^2^ of methotrexate was permeated from the nanoemulsion gel with a flux value of 2.078 ± 0.42 μg/cm^2^/h, according to permeation measurements. Finally, in vivo experiments on rabbit skin revealed that the increased skin penetration of methotrexate-loaded nanoemulsion gel was not due to structural alterations in intercellular lipid layers in the stratum corneum. In vivo antipsoriatic studies on rats revealed that MTX NEG produced a PASI decrease that was extremely similar and even better than the 91% reduction seen in the MTX tablet group. According to the pharmacokinetic profile, Cmax was 8.5 μg/mL, Tmax was 12 h, and t1/2 was 15.5 ± 2.37 h. These findings reinforce that MTX-NEG based on olive oil could be a possible treatment for psoriasis and could decrease the remission of psoriasis-like symptoms.

## 1. Introduction

Psoriasis is a recurrent chronic inflammatory disease with scaly, raised and red patches that is autoimmune in nature and multifactorial in character. According to epidemiological statistics, this disease affects 2.5 percent of the world’s population. This chronic illness affects both men and women equally and can strike at any age. It has an annual incidence rate of 50 to 140 new cases per million persons [1]. Psoriasis is thought to develop as a result of a complicated association between hereditary variables and environmental agents, including cigarette smoke, bacterial infection, oxidative stress, alcohol use and physical trauma. The treatment for psoriasis varies depending on the severity of the illness. Topical medicines are the first-line active therapies for psoriasis, and they are usually adequate to treat mild-to-moderate psoriasis [2]. Topical agents include coal tar [3], dithranol [4], vitamin D analogues, corticosteroids, retinoid, and keratolytic drugs such as salicylic acid [5]. When the results of topical therapy are inadequate or when the severity of psoriasis prevents the use of topical medicines, phototherapy and systemic therapy may be explored [6]. The current systemic treatments for psoriasis therapy include biological and non-biological treatment methods that are utilized as monotherapies or in combination regimens to treat mild-to-severe psoriasis [2]. Since 1972, methotrexate (MTX) has been used as a systemic therapy for moderate-to-severe psoriasis. Psoriasis is conventionally treated orally and parenterally with methotrexate. Because of its cytotoxic, immune-suppressive, and anti-inflammatory properties, the medication appears to provide therapeutic benefits in psoriasis [7,8]. Methotrexate is also said to increase cellular adenosine levels, which is thought to be responsible for its anti-inflammatory effects and clinical efficacy in psoriasis treatment.

About 30–90% of methotrexate patients experience side effects, which can range from moderate nausea to severe life-threatening pancytopenia, necessitating a dosage reduction or even the cessation of therapy. Methotrexate toxicity has been observed in the liver, bone marrow, and renal and pulmonary systems [9]. For the safe use of this medication in psoriasis, the FDA has issued a number of recommendations. Vital functions must be evaluated before commencing methotrexate medication, and monitoring must be performed at regular intervals during treatment, according to these guidelines.

There is a dire need for an efficient topical methotrexate administration method that will decrease systemic side effects associated with the drug. Easy and direct administration to localized psoriatic lesions, the absence of systemic adverse medication responses, application practicality, and non-invasiveness all contribute to the topical therapy being more convenient, resulting in increased patient compliance. As a result, efforts are being made to enhance methotrexate’s topical administration in order to decrease the drug’s systemic adverse effects in psoriasis. Although methotrexate is lipophilic, it is hydro soluble mainly at physiological pH and has a limited potential for passive diffusion through the skin; attempts to administer it topically have failed [10]. A transdermal matrix film of methotrexate was fabricated utilizing the solvent evaporation method and a blend of hydrophilic (HPMC) and hydrophobic polymer (EC) [11]. The transdermal flux does not reach the desired value in this case, and experimental data using the psoriatic model are unavailable. The transdermal iontophoretic administration of methotrexate was developed to improve its topical distribution [12].

To improve skin permeability, oleic-acid-loaded methotrexate deformable liposomes were developed [13]. It was reported that methotrexate-based nanostructured lipid carriers in conjunction with calcipotriol improve stratum corneum permeability [14]. However, all of these investigations are restricted to in vitro data, with no consideration given to the impact of these systems on genuine in vivo psoriatic models. Chitosan-decorated niosomal methotrexate gel was developed and tested in psoriatic patients. Because the reduction in the psoriatic area severity index (PASI) obtained was less than 75%, the method cannot be considered clinically effective [15]. As a result of the current literature, a novel carrier system for the effective topical administration of methotrexate in psoriasis is urgently needed. To achieve therapeutic efficacy without causing systemic toxicity, an optimal topical formulation of methotrexate should be able to offer appropriate skin penetration, as well as efficient drug localization to deep skin layers [16].

Nanodermatology is without a doubt a growing field of interest and significance, as well as promising a new era in the treatment and management of psoriasis [17]. The advent of nanoscale techniques allows for a prolonged drug release, while also protecting it against degradation. This is helpful because it maximizes therapeutic efficacy while reducing drug-related toxicities caused by clearance and overdosing. Furthermore, such therapeutical techniques minimize the frequency of medication administration, resulting in greater patient compliance [18,19].

Nanoemulsions are one of the techniques that is gaining a lot of interest for improving the skin penetration of hydrophobic drugs [20]. Nanoemulsions are kinetically stable and isotropic emulsion systems in which a tiny layer of emulsifier stabilizes the oil droplets carrying the medication. Owing to their prolonged physical stability, along with the tiny nanoscale of the droplets, the system can remain distributed without flocculation or coalescence, while also providing better thermodynamic stability. Nanoemulsion formulations are being researched intensively for their potential use as multifunctional nanocarriers in the field of pain and illness [21]. Nanoemulsions have been shown to improve the dissolution rates and bioavailability of medicines that are poorly water soluble, while reducing adverse effects. The system is suitable for topical administration due to its improved drug solubility, efficient drug loading capacity, superior thermodynamic stability, and permeation-enhancing activity without skin irritation [22,23]. However, because nanoemulsions have a low viscosity, they have a lower retentive capacity in the skin, which limits their usage in the pharmaceutical sector [24]. To overcome this limitation and provide an appropriate topical application, the viscosity must be raised by adding gelling agents such as carbopol 940, xanthan gum, sodium alginate, and so on [25]. This therapy inhibits the absorption of medication into the bloodstream and increases drug deposition in the skin, allowing for more effective action.

The goal of this study was to create a stable oil-in-water methotrexate-loaded nanoemulsion, which was then converted into a methotrexate nanogel using 1% sodium alginate for the relevant and effective dermal treatment of psoriasis with improved cutaneous methotrexate deposition and an enhanced local effect. Because the topical route of medication administration is less intrusive, simple, and pleasant, the effective use of a dermal preparation containing methotrexate improves patient compliance.

## 2. Materials and Methods

### 2.1. Materials

Methotrexate was generously gifted from Wilson Pharmaceuticals, Islamabad. Tween 80, PEG 400 and sodium alginate were purchased from Sigma Aldrich (St. Louis, MO, USA). Triethanolamine was purchased from Merck (Darmstadt, Germany). Olive oil was purchased commercially from the local market (Marhaba Industries, Lahore, Pakistan). All chemicals used in this study were of analytical grade and were used without further purification.

### 2.2. Animals

Male rabbits (2 ± 0.5 kg) were purchased from the local market to conduct in vitro skin permeation, as well as in vivo studies. Male Sprague Dawley rats (weighing 230 ± 20 g) were purchased from Peshawar University for in vivo antipsoriatic activity analysis. These two different animals were used in two different experiments, as mentioned. The animals were given free access to standard animal food, as well as water. All of the experimental procedures were approved by the Institutional Ethical Committee. Animals were kept apart during topical administration to prevent systemic drug entry by licking each other.

### 2.3. Preparation of Methotrexate O/W Nanoemulsion

The optimized formulation of methotrexate nanoemulsion (0.25% *w*/*w*) from our previous study was prepared by means of the high shear homogenization technique [26]. The homogenizer speed was set at 10,000 rpm for 15 min. Briefly, an oily phase containing olive oil, PEG 400, and methotrexate was magnetically stirred at 700 rpm for 1 h at 70 °C. Similarly, the aqueous phase was generated by continuously stirring Tween 80 into distilled water at 70 °C for 1 h. To achieve the final formulation, the oil phase was introduced drop-by-drop into the aqueous phase. To confirm the phase separation of the produced nanoemulsion, the optimal formulation was sonicated for 15 min before being placed at room temperature to investigate the thermodynamic stability of the nanoemulsion formulation.

### 2.4. Preparation of Methotrexate Nanoemulsion Gel

The optimized nanoemulsion formulation was distributed in different gel bases to maximize the local skin accumulation of the medication with the least amount of penetration in the blood. For the optimal nanoemulsion gel formulation, various concentrations of sodium alginate (0.5, 1, 1.5, 2 and 2.5%), HPMC K4M (1, 2 and 3%), and HPMC K100 (1, 2 and 3%) were employed as gelling agents. The sodium alginate (1%) gel was chosen for the production of the methotrexate nanoemulsion gel (MTX NEG) based on the physical appearance of a plain gel. The methotrexate-loaded nanoemulsion was combined with a 1% (*w*/*w*) sodium alginate gel to make a skin-suitable formulation. Briefly, sodium alginate was progressively added to distilled water while agitating continuously at 700 rpm for 2–3 h until a transparent dispersion was formed. To thoroughly hydrate and swell the alginate dispersion, it was left at room temperature for 3 h. The methotrexate-loaded nanoemulsion was combined with slight stirring into the sodium alginate dispersion. Finally, to produce a transparent gel, the mix was neutralized to a pH of 7.0 ± 0.5 by adding triethanolamine (4–5 drops) drop-by-drop. To allow entrapped air to escape, the prepared gel formulation was stored overnight [27,28]. For the production of plain methotrexate gel, a similar procedure was used. However, instead of a methotrexate-loaded nanoemulsion, a methotrexate solution (MTX Sol) in PBS (pH 7.4) was mixed into the sodium alginate dispersion. Table 1 shows the composition of the methotrexate-loaded nanoemulsion gel and the methotrexate plain gel.

### 2.5. Physico-Chemical and Rheological Characterization of Methotrexate Nanoemulsion Gel

The appearance, clarity, homogeneity, pH, drug content, and rheological properties of the developed methotrexate nanoemulsion gel and methotrexate plain gel were physicochemically analyzed. A visual inspection of gel compositions for clarity, appearance, and homogeneity was performed [29]. After diluting 1 g of gel in 100 mL of PBS, the concentration of methotrexate in the gel formulations was measured at 303 nm using a UV-Visible spectrophotometer (UV-1601 SCHIMADZU, Japan). To determine the pH of the gel formulation, 1 g of gel was dissolved in 25 mL of distilled water, followed by a pH measurement using a pH meter (Accumet meter 21039, Denver Instruments, USA) at 25 ± 1 °C. For instrument calibration, buffer solutions with known pH (3, 7, and 9) were employed. At room temperature, rheological characteristics of gel formulations were evaluated using a Brookfield viscometer (RVTD, USA) fitted with a UL-adapter [30]. With a #64 spindle, measurements were taken at a constant (40 rpm) as well as variable (10–120 rpm) shear speeds. The data were fitted to the appropriate rheological model based on the regression coefficient and rheograms of viscosity against shear rate were obtained [31].

### 2.6. Determination of Particle Size, Zeta Potential and Polydispersity Index

The zeta sizer Nano ZS 90 was used to determine these three characteristics of formulations of the methotrexate nanoemulsion gel and plain gel (Malvern Instruments, Worcestershire, UK). It comes with software version 6.34 and a laser with a wavelength of 635 nm and a detection angle of 90°. To achieve a homogeneous dispersion, blends of methotrexate gel formulations and deionized water were vortexed for 2 min. At a temperature of 25 ± 0.1 °C, the diluted formulations were examined in triplicates. The final results were given as a mean ± SD [32].

### 2.7. Morphology of the Methotrexate Nanoemulsion Gel Formulation

A scanning electron microscope (SEM, JSM 910, JEOL, Japan) was utilized to determine the shape and size of the methotrexate nanoemulsion gel formulation. The aqueous phase was removed from the produced formulation by centrifuging it at 10,000 rpm for 5 min. The sediment was combined with 3 drops of osmium tetra oxide (fixation medium) and maintained at 8 °C for 2 h. The material was diluted using a washing media that included 0.1 M phosphate buffer. The centrifugation and washing procedures were performed twice. Acetone was used to dehydrate the material. In the liquid sample cuvette, an aliquot of the formulation of the methotrexate nanogel was loaded on carbon film with 400 mesh copper grid. Then, at an accelerated voltage of 8.0 kV and a magnification of 10,000×, imaging of the specified sites was performed [33].

### 2.8. In Vitro Drug Release Determination

The release behavior of the methotrexate nanogel, plain gel and solution were evaluated using a Franz diffusion cell, employing a Tuffryn membrane at skin pH (5.5). This 0.45-μm pore membrane served as a partitioning medium between the donor and receptor compartments of the Franz diffusion cell (Perme gear, Inc. No: 4G-01-00-15-12; Mumbai, India: diffusion area = 1.767 cm^2^). About 7.0 mL of fresh phosphate buffer (pH 5.5), maintained at 32 ± 2 °C was filled into the receptor compartment to mimic skin fluid. At regular intervals, 1 mL samples were taken from the receptor compartment. The same amount of phosphate buffer was substituted to maintain the sink conditions. A UV-Vis spectrophotometer (UV-1601 SHIMADZU, Japan) was used to examine the samples. The mean ± SD of the triplicate results was displayed. The data were plotted as time versus concentration on a graph. The drug release data were analyzed using a power law kinetic model, as indicated in the equation below [34].

Power Law

Mt/M_∞_ = Ktn (1)

where Mt/M_∞_ represents a fraction of the drug released after time t, K is the rate constant, and n represents exponential release value.

When *n* = 0.5, then release occurs through the quasi–Fickian diffusion mechanism. When *n* > 0.5, then the release mechanism is anomalous, with non-Fickian diffusion, and *n* = 1 shows a zero-order release mechanism or case II transport.

### 2.9. In Vitro Skin Permeation of Methotrexate Gel Formulations

The technique outlined in our earlier work was used to accomplish in vitro skin penetration of methotrexate gel formulations [26]. After being sacrificed via an intravenous injection of sodium phenobarbital ketamine, followed by cervical dislocation, the skin was meticulously removed from the abdomen region of male rabbits (2 ± 0.5 kg). The skin was rigorously cleaned of underlying lipids and connective tissues, then rinsed in saline, wrapped in aluminum foil, and kept at −20 °C until needed. Frozen skin was thawed at room temperature for 30 min before permeation tests.

A Franz diffusion cell (Perme gear, Inc. No: 4G-01-00-15-12; India) was used to conduct in vitro skin permeation investigations. It was equipped with vertical cells, heating circulation, magnetic stirring, as well as temperature control systems. The cell had an effective permeation area (1.77 cm^2^) with a receptor cell volume of 7 mL. About 7 mL of PBS (pH 7.4) was added to the receptor compartment, which was constantly stirred at 500 rpm. Throughout the experiment, the temperature of the receptor compartment was kept constant at 37 ± 0.5 °C. Between the donor and receptor compartments of the diffusion cell, rabbit skin was affixed. Under an open hydration procedure and non-occlusive application, the donor compartment was filled with 500 mg of the gel formulation [35,36]. At predefined periods (0.5, 1, 2, 4, 8, 12, 16, 20, and 24 h), samples (0.5 mL) were removed from the diffusion cell’s sampling port, and an equivalent volume of new diffusion medium was kept at the same temperature and was immediately introduced to the receptor compartment. The MTX content of the obtained samples was determined using a UV–vis spectrophotometer (UV-1601 SHIMADZU, Hadano, Japan) set at 303 nm. A graph was constructed by plotting the cumulative amount of MTX permeated per unit area versus time period, and flux was determined using the slope of the curve. The magnitude of the formulation flux, as well as the initial MTX concentration in the donor compartment could be employed to calculate the permeability coefficient, whereas the enhancement ratio was generated via the division of the flux value of the MTX nanogel to that of the MTX solution.

### 2.10. Skin Drug Retention Analysis

Upon completion of the permeation experiment, the skin mounted in the Franz diffusion cell was meticulously removed. To extract the drug retained, the skin was thoroughly cleaned with phosphate buffer solution. Before cutting it into small pieces (1 cm^2^), the skin was tap dried with soft tissue paper and stirred in phosphate buffer solution of pH 7.4 overnight. The samples were then filtered through a 0.45 μm cellulose acetate filter and examined on a UV visible spectrophotometer at a 303 nm wavelength the next day [37]. The experiment was repeated three times, with the results averaged.

### 2.11. Mechanism of Drug Retention

With the ultimate goal of increasing methotrexate skin drug accumulation, the skin drug retention mechanism of the improved formulation was investigated using ATR-FTIR on treated skin samples. Briefly, treated skin samples of the nanoemulsion gel, the gel base, as well as the nanoemulsion components, were exposed to vibrational spectroscopic examination by pressing the skin against a zinc selenide crystal to guarantee high sensitivity and intimate contact. Both the epidermis and the dermis were scanned at wave numbers ranging from 675 to 4000 cm^−1^. The typical peaks were reported. At least three replicates were performed, and the findings were averaged.

### 2.12. Anti-Psoriatic Activity Studies

We employed an imiquimod (IMQ)-generated psoriatic animal model to test anti-psoriatic effectiveness. A pilot study in our laboratory was used to standardize the model. Male Sprague Dawley rats were split into four groups, as illustrated in Table 2. The animals were housed in pathogen-free environments and given unrestricted access to food and water. The hair on the animals’ backs was removed using an electric clipper, and the shaved region was treated with 62.5 mg of IMQ (5%) for 7 days to see if psoriatic-type skin lesions developed. Following the onset of psoriasis, IMQ was used occasionally (three days at a three-day interval) for another three weeks in order to maintain psoriasis. The formulations were applied daily to IMQ-treated regions to see if psoriasis improved to evaluate efficacy. The dosage of an oral methotrexate tablet given weekly (MTX tablet) was 5.143 mg/kg (mouse dose equal to 25 mg clinical dose for an average 60 kg human). For delivery, a commercially available tablet of 15 mg dissolved in 30 mL of PBS and delivered through oral gavage tubes in a volume capable of transferring the required dosage. The clinical psoriasis area and severity index (PASI) score measures were used to evaluate the extent of skin irritation and inflammation during the therapy period. The thickening, erythema and scaling of the animal’s skin were evaluated separately on a scale of 0 to 4 for the PASI score determination, where 0, none; 1, minor; 2, moderate; 3, noticeable; 4, severe. During the treatment period, scoring was done every 24 h, and the total PASI was computed [38,39]. The percentage reduction in PASI at the end of the trial was compared to PASI on the 7th day and the induction of psoriasis.

### 2.13. HPLC Analysis of Methotrexate

Methotrexate was quantified using a high-performance liquid chromatography system (Agilent 1269, Agilent Technologies, Santa Clara, CA, USA). A C18 column (4.6 1003.5 mm, Zorbax C18, Agilent, USA) was utilized in the experiment. The mobile phase was made up of a buffer (0.2 M Na2HPO4 and 0.2 M citric acid in a 2:1 ratio) at pH 6.0 and acetonitrile in a 90:10 (*v*/*v*) ratio. The mobile phase was maintained at a constant flow rate of 1 mL/min at 25 °C. By injecting 20 μL of each sample, all of the samples were examined. The wavelength of the UV detector was adjusted at 303 nm [1].

### 2.14. In Vivo Analysis

Healthy male albino rabbits, used as test animals, were purchased from a local market. They were kept at room temperature (25 ± 2 °C) with a relative humidity of 55 ± 5% and were given normal access to food and drink for seven days while acclimating. All animal procedures followed the institution’s ethical norms, as well as international standards. A ketamine-xylazine injection was administered intramuscularly to anesthetize the rabbits. The back was shaved and cleaned with an ethanol swab in an area of around 2–3 cm^2^.

All of the animals were separated into two groups, each with six animals: group A and group B. Group A (the control group) was given aqueous methotrexate solution, whereas group B (the test group) was given an optimized nanogel formulation. At regular intervals, 0.5 mL of blood was taken and centrifuged to separate plasma. Adding methanol to plasma and vortexing for 10 min precipitated plasma proteins, which were then centrifuged for 30 min at 5000 rpm. The clear supernatant was subjected to filtration, followed by its dissolution in 0.5 mL of HPLC mobile phase (0.2 M Na2HPO4 and 0.2 M citric acid in a 2:1 ratio) at pH 6.0 and acetonitrile in 90:10 (*v*/*v*) and evaluated via HPLC for drug contents that were permeated.

An overdose IV injection of sodium pentobarbital was administered to sacrifice the rabbits when the permeation experiment was over. Skin excision into small pieces was performed meticulously, followed by saline washing and tap drying. The skin was swirled in distilled water overnight to remove methotrexate, then filtered and analyzed using HPLC to determine the residual drug concentration [40].

### 2.15. Stability Studies

Nanoemulsion gels have the ability to improve the physical and chemical stability of drugs, so formulations of nanoemulsion gels have been subjected to stability testing. In order to determine the stability studies, the formulations were stored under various storage settings. For 90 days, the formulations were kept in the refrigerator (2–8 °C) by sealing in a glass container, at ambient temperature (25 ± 5 °C), and at accelerated temperature (40 °C and 65% RH). After 1, 2, and 3 months, the formulations were examined for any changes in color or clarity, particle size, phase separation, zeta potential, pH, polydispersity index, and drug content [37].

### 2.16. Statistical Analysis

All of the tests were carried out in triplicate. The data were presented as mean ± SD. SPSS software version 18 was used to analyze the statistical data (SPSS Inc., Chicago, IL, USA). For statistical analysis, Student’s *t*-test or ANOVA were employed. A *p*-value < 0.05 was deemed significant.

## 3. Results and Discussion

### 3.1. Methotrexate-Loaded Nanoemulsion Gel Formulation

Because of the excellent skin penetration results of the optimized MTX nanoemulsion formulation from our earlier study, it was also chosen for further studies [26]. Many studies were conducted to find an appropriate vehicle for topically applying the methotrexate-loaded nanoemulsion. Because of its greater storage stability, outstanding bioadhesive characteristics, non-irritating behavior, and the capacity to produce clear and pharmaceutically elegant gels, 1% sodium alginate (*w*/*w*) was chosen as the gel-forming base [41]. Preliminary tests were carried out to establish the suitable concentration of sodium alginate. It was necessary to obtain optimum gel viscosity for application to the skin, while avoiding the possibility of the formulation being drained out.

### 3.2. Physicochemical and Rheological Characterization of Methotrexate Nanoemulsion Gel

**Figure 1:**
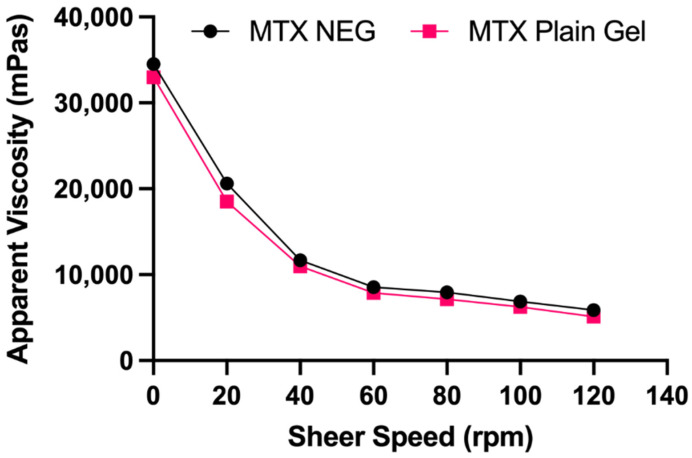
Flow curves of MTX NEG and MTX plain gel. Data are expressed as mean ± SD (*n* = 3).

Table 3 lists the physicochemical and rheological characteristics of gel formulations. All formulations were homogeneous, clear, and had a high drug content (92.35 ± 0.6% and 97.5 ± 0.55%). The MTX nanogel seemed somewhat opaque in comparison to the transparent MTX plain gel because of the presence of dispersed vesicles. The pH of the prepared gels was in the skin’s pH range, which is appropriate for topical administration without any irritation to the skin [42]. The rheological characteristics of a gel are significant because they influence the ease of topical application, as well as subsequent adherence and retention at the application site. Contrary to MTX plain gel (10,987 mPa s), greater viscosity was exhibited by MTX NEG (11,674 mPa s) at a constant shear speed (40 rpm). The responsible factor might be the nanoemulsion which was incorporated into the gel base [43]. Plotting the apparent viscosity against the corresponding shear speed yielded the flow curves for gel compositions (Figure 1). The flow curves indicated that gel formulations exhibited a non-Newtonian, pseudoplastic (shear thinning) flow behavior, as shown by a drop in viscosity as shear speed increased [44,45]. Pseudoplastic flow characteristics are advantageous in topical preparations because they guarantee maximal coverage when applied [41].

### 3.3. Size, Size Distribution and Zeta Potential

The values of particle size, PDI, and zeta potential are shown in Table 4 for both plain and drug-loaded formulations. Methotrexate-loaded nanoemulsion gel has a globule size of 202.6 ± 11.59 nm and a PDI of 0.233 ± 0.01. The particle size of a nanoemulsion gel determines the drug’s absorption and bioavailability. Because small particle size produces a large surface area, the drug release into the aqueous media is increased, leading to increased drug absorption. It has been proposed that the incorporation of drug particles results in their interaction with the system’s microstructure, lowering globule size, especially if the drug exhibits an amphiphilic nature. Within the formulation, PDI guarantees globule size consistency. If the PDI value is < 0.5, the formulation consists of a homogenous and uniform globule size. A smaller globule size plays a very important role in the topical application of the formulation because it increases skin permeability. The zeta potential value of the methotrexate-loaded nanoemulsion gel was optimum (−14.2 ± 4.42 mV), indicating the physical stability of the system. Electrostatic repulsion between particles is represented by the zeta value. Zeta potential values > ±30 mV ensure the stability of preparations having no aggregation [46]. The electrostatic repulsive interactions between oil globules were validated by higher zeta potential values, which prevented coalescence and an adequately stable and uniform dispersion resulted. In this case, the free fatty acids provide the oil globules with a negative charge, and the adsorption of hydroxyl ions from water onto the oil–water interface confers to the system an overall negative charge.

### 3.4. Scanning Electron Microscopy (SEM)

**Figure 2:**
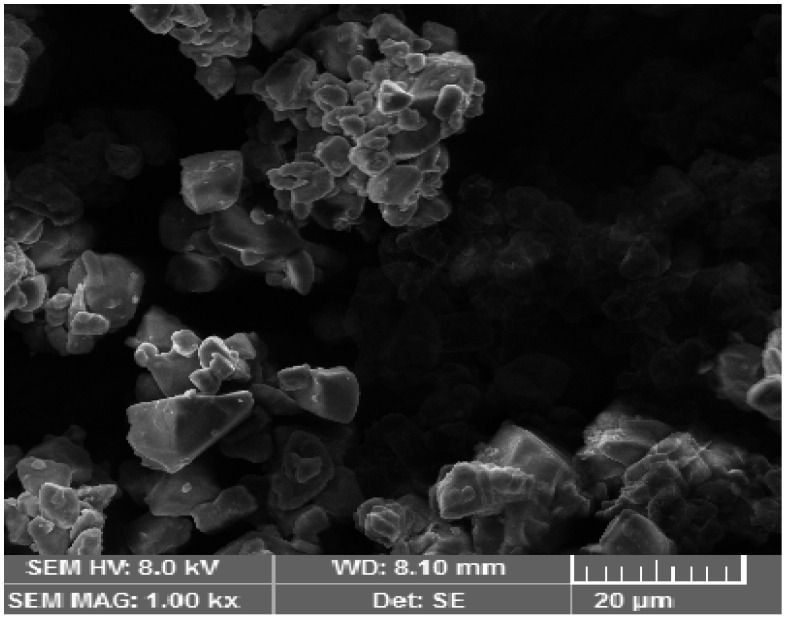
SEM image of the methotrexate-loaded nanoemulsion gel optimized formulation.

SEM was used to obtain important morphological information. Nanoemulsion particles were evenly dispersed in the polymeric gel base and exhibited a well-defined spherical shape. The porous nature of the alginate gel had embedded nanoemulsion particles, forming the gel layer barrier, providing the controlled release of the drug from the formulation. The spherical shape is beneficial because it squeezes easily through minute skin pores, allowing for increased system permeability. The SEM image is shown below in Figure 2.

### 3.5. In Vitro Drug Release Studies

**Figure 3:**
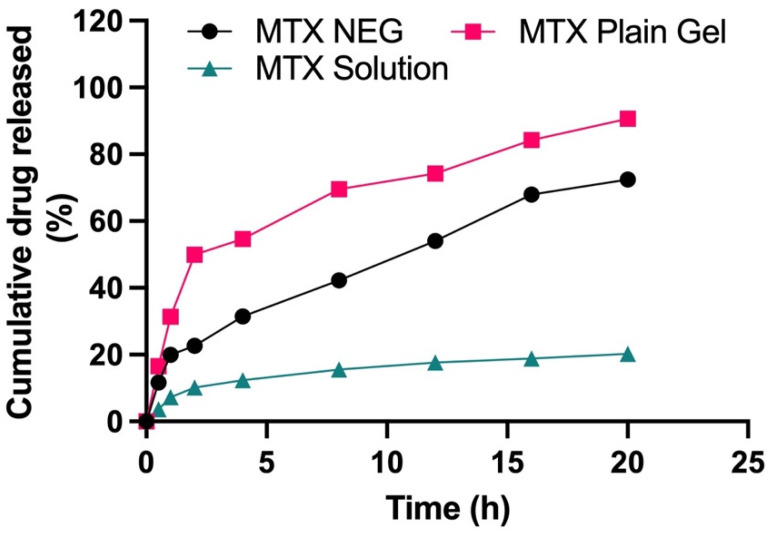
Cumulative % age drug release from MTX solution, MTX plain gel, and optimized MTX NEG formulations (*p* < 0.05, MTX plain gel vs. MTX NEG vs. MTX sol).

The drug product is incorporated into nanocarrier systems in either of the two forms—dispersed or dissolved [47]. As a result, drug solubility in the lipid matrix is a critical component in regulating the drug release from nanocarriers. To fulfil the criterion, in-vitro drug release experiments were used to evaluate the drug release profile. A UV spectrophotometer with a wavelength of 303 nm was utilized for this purpose. To examine the in vitro release behavior of different formulations, the Franz diffusion cell was employed. The in vitro drug release was measured under conditions mimicking physiological skin conditions. A receptor medium, consisting of phosphate buffer, having a pH of 5.5 and kept at a temperature of 32 ± 0.5 °C, was utilized in this study. Methotrexate solution, methotrexate plain gel and methotrexate nanogel (MTX–NEG) were studied in comparison, and the findings are given in Figure 3. Methotrexate plain gel showed a fast initial release of the drug in the first 2 h, followed by a 20-h period of prolonged release. Superficial entrapment of the drug might be the cause of the initial burst release from the plain gel of the drug. In nanodermatology, the demonstrated sustained and prolonged drug release behavior is of significant interest. Hence, it can be inferred that the intended topical administration of the drug may be determined from this type of drug release behavior. As a result, the initial fast and then slow release of methotrexate reduces the loss of the drug that may take place as a result of everyday activities. Changing clothing and sweating are two examples of activities. This promotes a better skin administration due to the moisturizing/occlusive properties of the nanoemulsions, as well as a well-maintained slow and extended drug release. At pH 5.5, methotrexate plain gel releases around 90.63% of the medication after 20 h. The drug release characteristics, in descending order, were MTX plain gel > MTX-NEG > MTX solution (ANOVA, *p* < 0.05). Interactions between the drug and the surfactants, as well as partitioning of the drug between the aqueous and oil phases, are all regulating variables for the drug release. The globules must possess a smaller size, and a greater surface area, for the effective drug release from the nanoemulsion gel. The nanosize of the formulation increases the rate of MTX dissolution into the aqueous phase. Solubility, as well as the drug release, are also improved. The release occurs in a regulated manner. In the case of the MTX plain gel formulation, there is the absence of a burst release pattern, except there is an initial fast release within the first 2 h. A slow release is an ideal parameter in chronic conditions such as psoriasis, whereas a fast or burst release is not. A burst release also causes toxicity, since the medication is released and absorbed more quickly in inflamed psoriatic lesions [48]. In order to achieve a delayed release of the active moiety, as well as better drug retention characteristics, the optimized nanoemulsion formulation was transformed into gel form. According to kinetic data analysis, shown in Table 5, the release mechanism for methotrexate solution was zero-order (R^2^ value of 0.8708) at pH 5.5. At pH 5.5, the Fickian diffusion pattern is followed by the MTX plain gel formulation, with an R^2^ value of 0.9377. The Korsemeyer Peppas model fitting depicted the non-Fickian diffusion mechanism of the drug release with an *n*-value of 0.515 at pH 5.5.

### 3.6. In Vitro Skin Permeation of Methotrexate Gel Formulations

**Figure 4:**
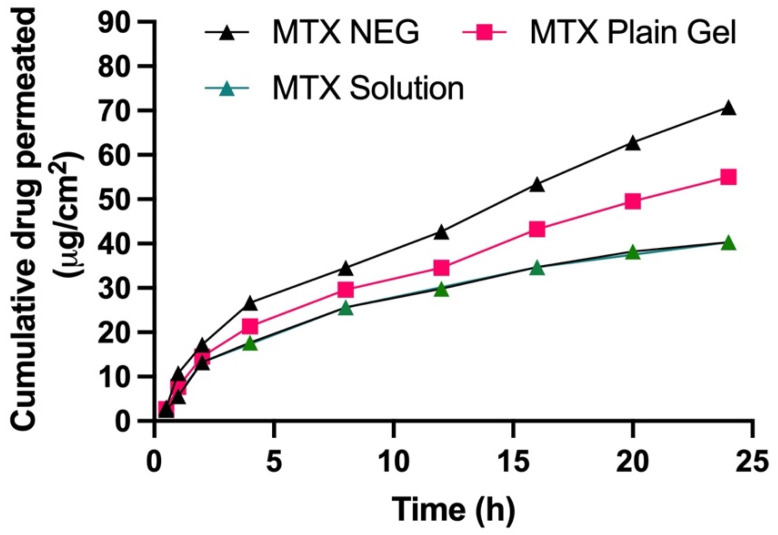
Skin permeation profile of MTX sol, MTX NEG, and MTX plain gel across rabbit skin for 24 h. Data are expressed as mean ± SD (*n* = 3).

Using a Franz diffusion cell, the in vitro skin penetration capability of MTX NEG was assessed by employing rabbit skin and was compared to that of the MTX solution and the MTX plain gel. Figure 4 depicts the skin penetration patterns of gel formulations after 24 h. Permeation profiles were used to compute the total quantity of MTX that penetrated after 24 h, as well as the permeation flux and enhancement ratio, which are presented in Table 6. The MTX permeation from gel formulations was sluggish at first, but after 2 h, there were significant variations in the total quantity of MTX permeated. After 24 h, the cumulative quantity of MTX permeated from MTX NEG (70.78 ± 5.8 μg/cm^2^) was substantially greater than that penetrated from the MTX solution (40.31 ± 5.3 μg/cm^2^) and MTX-plain-gel (54.98 ± 4.9 μg/cm^2^) (ANOVA, *p* < 0.01). Furthermore, as compared to the MTX solution (1.092 ± 0.04 μg/cm^2^/h) and MTX-plain-gel (1.614 ± 0.39 μg/cm^2^/h), permeation flux of MTX from MTX NEG (2.078 ± 0.42 μg/cm^2^/h) was 1.903 and 1.287 times greater, respectively. MTX NEG > MTX plain gel > MTX solution was the order of the improvement in the skin permeation order. The potential of particles of the MTX nanoemulsion to compress themselves through the comparatively tiny pores of the skin explains the greater skin penetration of MTX from MTX NEG. Furthermore, poor MTX-plain-gel permeation might be attributed to MTX’s inherently low permeability due to its lower partition coefficient and ionization potential under physiological circumstances [49].

### 3.7. Drug Retention Analysis

**Figure 5:**
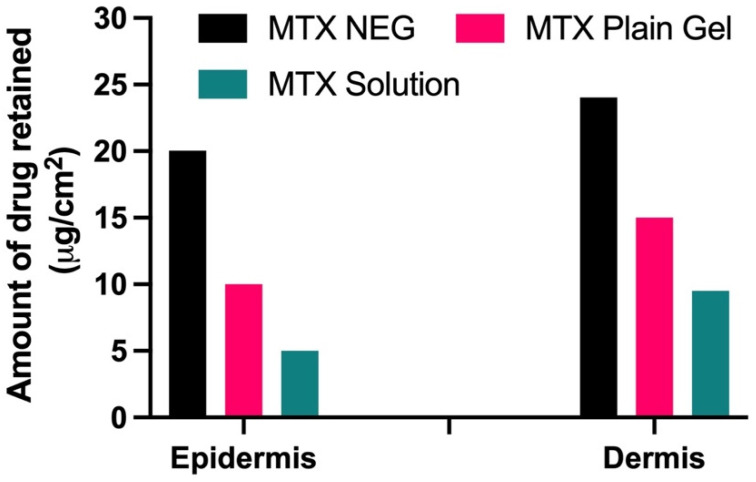
Amount of drug retained (µg/cm^2^) in epidermis and dermis layers of the skin (*p* < 0.01 vs. MTX plain gel and MTX sol).

Drug retention tests revealed that when MTX-NEG formulations were compared to MTX plain gel and the control drug solution, the drug was kept more in the deeper epidermis and dermis layers (ANOVA, *p* < 0.01). Because of the increased association between keratinocytes and the nanosized formulation, there is more retention. In addition, the use of Tween 80 improves MTX deposition in both the epidermis and dermis [50]. Because the dermis is thicker than the epidermis, medication retention will be higher in the dermis than in the epidermis, according to skin anatomy. Because both layers are primarily influenced by psoriasis, the higher drug retention in each of these layers is deemed noteworthy (Figure 5).

### 3.8. Impact of Formulations on Skin Structure

An ATR-FTIR analysis was undertaken to examine the effects of the MTX nanogel formulation on the lipid display of the stratum corneum. This was executed by the interaction of the formulation with the rabbit epidermis for a period of 4 h. The major permeability barrier in transdermal administration is keratinized corneocytes, which are flat and cushioned in the intercellular lipoid region of the stratum corneum. Alcohols, terpenes, fatty acids and surfactants might be utilized in optimal transdermal formulations to decrease the barrier function of the stratum corneum, since these substances not only permeate into the intercellular areas but also promote fluidization and instability of lipid barriers [51]. Because of its notable penetration characteristics, as well as its suitability as a topical formulation, the optimized olive oil methotrexate nanogel was chosen for this investigation.

**Figure 6:**
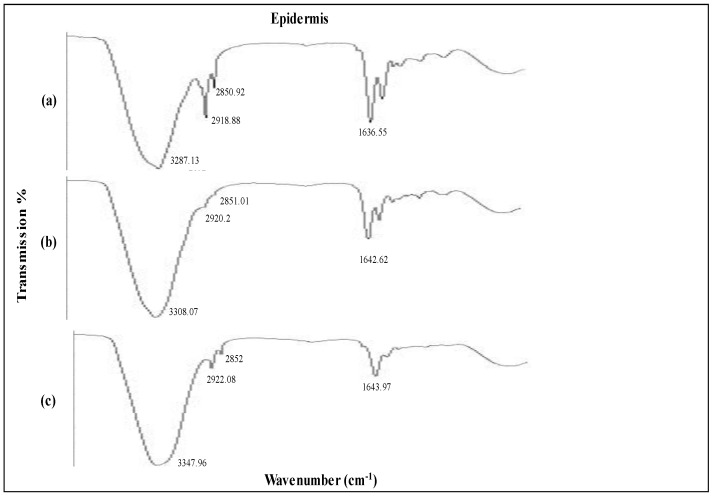
IR spectroscopy images of epidermis (**a**) untreated skin and skin loaded with (**b**) MTX plain gel and (**c**) MTX NEG.

**Figure 7:**
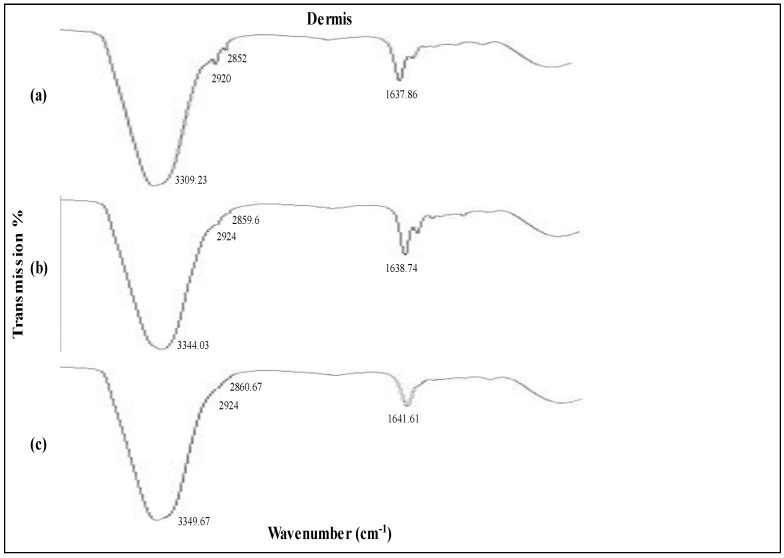
IR spectroscopy images of dermis (**a**) untreated skin and skin loaded with (**b**) MTX plain gel and (**c**) MTX NEG.

The FTIR spectra of the formulations under investigation are shown in Figure 6 and Figure 7. The untreated skin’s distinctive peaks were 3237.13 cm^−1^, 2918.28 cm^−1^, 2850.92 cm^−1^, and 1636.55 cm^−1^. Between 2800 and 3200 nm, the absorption bands corresponding to the amine, aromatic hydrocarbon, and alkane groups. Normal polymeric hydroxyl stretching is attributed to the first band. C=H may be attributed to the wavenumbers recorded at 2918.18 and 2850.92. The band at 1636.55 corresponds to the C=C stretching of an alkenyl functional group [52].

A higher wavenumber shift was observed in the case of untreated skin epidermis upon exposure to the MTX NEG formulation. The FTIR peaks at 3287.13cm^−1^ corresponding to an O-H and/or N-H moiety of both lipids and/or proteins were moved to higher wavenumbers at 3308.07cm^−1^, 2920.2cm^−1^ and 3347.96cm^−1^, 2922.08cm^−1^. Similarly, amide 1 proteins were also moved to higher wavenumbers at 1642.62cm^−1^ and 1643.97cm^−1^ due to the interaction of the MTX NEG formulation and untreated skin. The findings clearly indicate that MTX NEG efficiently fluidized the skin’s lipid and protein by enhancing membrane permeability, resulting in improved formulation permeation and retention.

ATR-FTIR analysis revealed that MTX NEG-treated skin dermis switched to increased wavenumbers at 3344.03cm^−1^ and 3349.67cm^−1^, corresponding to O-H and/or N-H bonds of ceramide, keratin, and/or lipids. In addition, formulation-treated skin also showed a higher wavenumber of the ATR-FTIR peak at 2920cm^−1^ and 2852cm^−1^ for the untreated dermis. It was reported that when MTX NEG interacts with keratin and/or polar ceramide regions and lipid components in the stratum corneum, the skin’s hydrogen bond strength is reduced, resulting in efficient skin fluidization [53].

The findings demonstrate that the optimized MTX NEG formulation effectively fluidized both the epidermis and dermis of skin, potentially increasing drug permeability and retention. The implication of tween 80 and PEG 400, on the other hand, significantly enhanced these effects.

### 3.9. Anti-Psoriatic Activity Studies

Psoriasis was induced by applying imiquimod (IMQ) to the skin for seven days in a row. On the back skin of mice, daily treatment of IMQ resulted in inflammatory scaly skin lesions that resembled plaque pattern psoriasis.

PASI is a test that is widely used to assess the clinical effectiveness of anti-psoriatic treatment. PASI assesses lesions’ severity, focusing on the area involved. We assessed the lesions’ severity daily during in vivo investigations by visually evaluating the thickness, erythema, and inflammatory scales. these characteristics were summed up to generate the cumulative PASI score. The difference between the PASI at therapy termination and the PASI on the 7th day of psoriasis induction is computed and displayed in Table 7. Only an 8.29% PASI decrease, evident by the retention of psoriatic lesions due to imiquimod therapy, might be attributed to the model’s self-healing behavior. The anti-oxidant and anti-inflammatory properties of olive oil are thought to be responsible for the oil’s 46% decrease in PASI. MTX NEG produced a PASI decrease that was extremely similar and even better than the 91% reduction seen in the MTX tablet group.

On the 14th day, the PASI score began to decline in these groups of animals, with inflammation completely disappearing on the 28th day. A PASI decrease of 75% or more is required to consider an anti-psoriatic treatment clinically successful. On this premise, MTX NEG and MTX tablets can be considered effective anti-psoriatic medications. The therapeutic effectiveness of methotrexate tablets in the treatment of psoriasis has been extensively established. Methotrexate has clinical effectiveness in psoriasis because of its anti-inflammatory, cytotoxic, and immune suppressive potential [54]. The improved skin permeability and drug retention ascribed by the formulation may account for the efficacy reported with the topically administered nanoemulsion gel. Because of the enhanced skin penetration, the medication will be able to work systemically as it occurred in the oral tablet group. The drug’s enhanced skin retention allows it to operate locally in deep skin layers via cytotoxic and immune-suppressant pathways, which may explain why it has a little higher effectiveness than oral methotrexate tablets. In addition, the antioxidant and anti-inflammatory properties of the olive oil included in the composition give considerable benefits. The MTX plain gel reduced PASI by 61%, which might also be attributed to an increase in the anti-inflammatory action of the olive oil inside the nanosystem.

### 3.10. Plasma Concentration of Methotrexate

**Figure 8:**
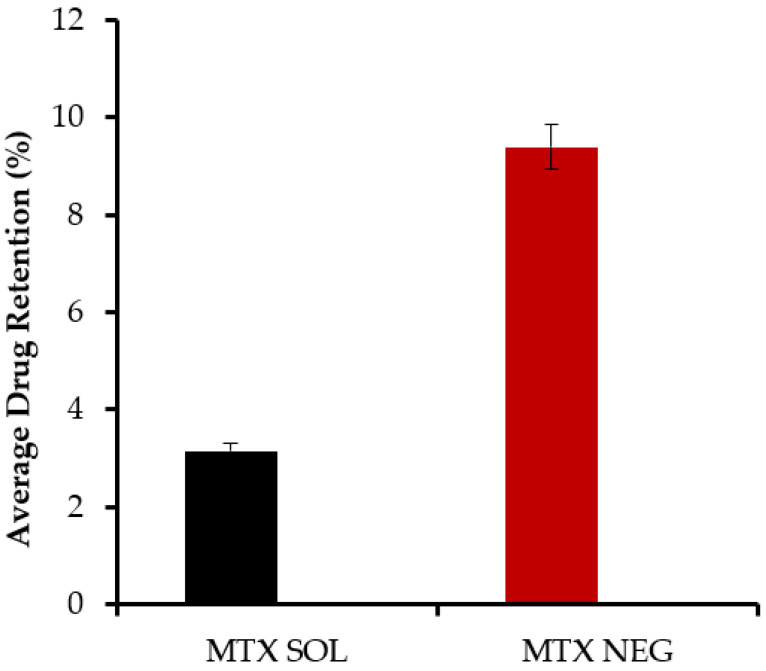
Methotrexate concentrations in the skin from methotrexate solution and methotrexate nanoemulsion gel, respectively, calculated from in vivo studies (*p* < 0.001).

**Figure 9:**
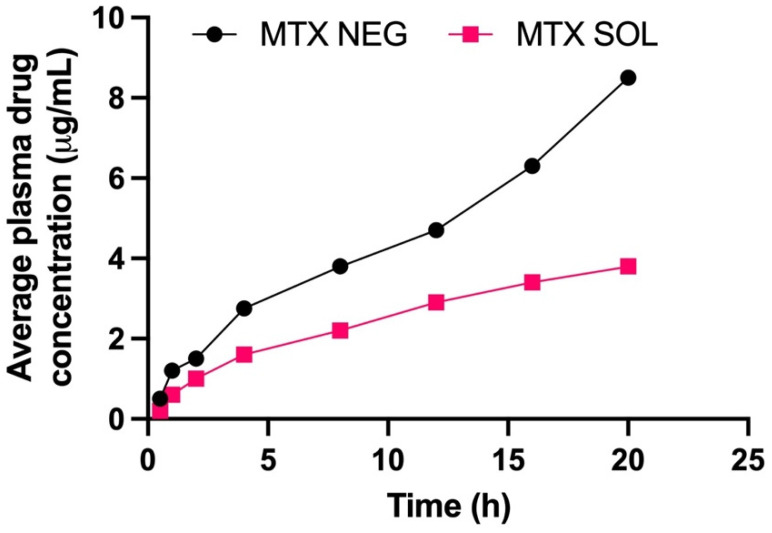
Methotrexate concentrations in the plasma from methotrexate solution and methotrexate nanoemulsion gel, respectively (*p* < 0.001).

Table 8 summarizes the pharmacokinetic variables of MTX-NEG. The MTX-NEG topical formulation had a plasma level of 8.5 ± 0.78 µg/mL, as per the plasma drug concentration measurements. The enhanced MTX permeability on the topical application is used to determine the drug’s penetration into the systemic circulation in the scenario of MTX-NEG therapy. Because methotrexate’s clinical efficacy is somewhat driven by a systemic pathway in the case of psoriasis, the systemic entry of a topically applied medication is helpful. In the case of MTX-NEG, low serum concentrations reduce drug accumulation in key systems and prevent related systemic toxicities. However, long-term consumption of MTX oral tablets has been linked to these side effects [55]. The computed values show that methotrexate’s biological half-life (t1/2) in rabbits is increased from 4–10 h^−1^ to 15.5 ± 2.26 h^−1^ (transdermal). As a result, the medication delivered by MTX-NEG will stay in the body for a longer duration, producing a sustained effect. Methotrexate has a substantially lower elimination rate constant (0.032 ± 0.003 h^−1^) and an extended mean residence time (11.86 ± 0.69 h), both of which support the drug’s prolonged activity from the nanogel formulation. The enhanced bioavailability of the medication is also shown by the high AUC value obtained with the nanogel formulation. This might be linked to the hepatic first-pass effects being bypassed and stomach degradation being avoided (Figure 8 and Figure 9).

### 3.11. Stability Determination

Stability tests were carried out to assess the stability of the developed formulation over time under different environmental conditions (humidity, temperature, and light). As a result, determining a suitable storage environment for the MTX nanoemulsion gel is an indirect approach. For three months, the developed formulation was monitored for changes in droplet size, PDI, surface charge, and pH, under the storage conditions given in Table 9. There was no turbidity or phase separation after 3 months of storage at the stated conditions, indicating that the formulation is physically stable. The stored methotrexate-loaded nanoemulsion gel and the freshly developed formulation had a similar surface charge and pH. However, globule size increased negligibly after 3 months’ duration when stored at ambient and accelerated pre-defined official compendial specified conditions. The contributing factor for this effect might be the increased kinetic energy of the globules that led to random globule collision and aggregation [48]. However, the globule sizes of the formulation and that of the freshly prepared nanoemulsion gel were nearly identical (18.27 ± 5.78 nm), having PDI < 0.3 at refrigerated temperatures over three months, indicating substantial formulation stability in line with the method employed for its production. Because the changes in particle size and other characteristics of the developed formulation and the freshly prepared formulation were minimal, these stability test findings indicated that the methotrexate-loaded nanoemulsion gel should be subjected to chilled storage [56].

## 4. Conclusions

For topical application in psoriasis, an olive oil-based methotrexate nanoemulsion gel was formulated. The developed formulation has optimal properties for the efficient topical administration of methotrexate with improved skin penetration and retention, making it a promising alternative to oral methotrexate tablets. Superior anti-psoriatic effectiveness with a reduced serum accumulation, achieved with the topical use of a nanoemulsion gel, is predicted to enhance patient compliance, while reducing systemic toxicity.

## Figures and Tables

**Table 1:** Composition of MTX NEG and MTX plain gel (*w*/*w*, %).

Components	MTX NEG	MTX Plain Gel
Sodium Alginate	1	1
Triethanolamine	1	1
MTX Nanoemulsion	50	_______
MTX Solution	______	50
Distilled Water	48	48

MTX: methotrexate, NEG: nanoemulsion gel.

**Table 2:** Grouping of animals in anti-psoriatic studies.

Groups	No. of Animals	Treatments	Route	Duration of Study
1	6	Normal control		1 month
2	6	Imiquimod cream	Topical	1 month
3	6	MTX plain gel	Topical	1 month
4	6	MTX NEG	Topical	1 month
5	6	Olive oil	Topical	1 month
6	6	MTX tablet	Oral	1 month

**Table 3:** Physicochemical and rheological parameters of nanogels.

Character	MTX NEG	MTX Plain Gel
Physical Appearance	Slightly opaque	Transparent
Clarity	Yes	Yes
pH	6.97 ± 0.19	7.01 ± 0.12
Homogeneity	Good	Good
Drug Content (%)	92.35 ± 0.6	97.5 ± 0.55
Viscosity (mPa s)	11,674 ± 121.15	10,987 ± 77.76
Flow Behavior	Non-Newtonian	Non-Newtonian

Data are expressed as mean ± SD (*n* = 3).

**Table 4:** Characterization of MTX nanogel formulations.

Formulation	Particle Size (nm)	PDI	Zeta Potential (mV)	EE%
MTX plain gel	173.1 ± 12.35	0.310 ± 0.42	−8.59 ± 6.94	58.45 ± 4.63
MTX NEG	202.6 ± 11.59	0.233 ± 0.01	−14.2 ± 4.42	76.57 ± 2.48

Note = Data are expressed as mean ± SD (*n* = 3).

**Table 5:** Release kinetics of prepared formulations.

Formulations	Power Law Kinetic Model			
	K ± SD	R^2^	*n*	Release Mechanism
MTX SOL	0.30 ± 0.155	0.8708	0.413	Fickian Diffusion
MTX Plain Gel	0.001 ± 0.00189	0.9377	0.427	Fickian Diffusion
MTX NEG	0.005± 0.0120	0.9688	0.515	Anomalous Non-Fickian Diffusion

**Table 6:** Skin permeation parameters of MTX NEG, MTX plain gel, and MTX sol.

Formulation	Cumulative Amount Permeated for 24 h (µg/cm^2^)	Permeation Flux (µg/cm^2^/h)	Enhancement Ratio
MTX NEG	70.78 ± 5.8	2.078 ± 0.42	1.903
MTX plain gel	54.98 ± 4.9	1.614 ± 0.39	1.478
MTX SOL	40.31 ± 5.3	1.092 ± 0.04	1.00

Note = Data are expressed as mean ± SD (*n* = 3) *p* < 0.01 vs. MTX plain gel and MTX sol.

**Table 7:** PASI decrease % after 28 days.

Treatment Exposure	PASI		% Decrease in PASI
	Induction of Psoriasis	Therapy Termination	
Imiquimod cream	7.72 ± 0.35	7.08 ± 0.89	8.29 ± 0.97
MTX plain gel	7.63 ± 1.83	2.95 ± 0.73	61.33 ± 3.28
MTX NEG	7.54 ± 1.43	0.38 ± 0.54	94.96 ± 4.99
Olive oil	7.59 ± 0.44	4.10 ± 0.21	45.98 ± 4.41
MTX tablet	7.51 ± 0.64	0.62 ± 1.41	91.74 ± 2.46

**Table 8:** Pharmacokinetic parameters of methotrexate from methotrexate nanogel upon administration on skin in rabbits up to 24 h.

Parameters	Methotrexate Nanoemulsion Gel (MTX-NEG)
Cmax (µgm/mL)	8.5 ± 0.78
Tmax (h)	12
K (h^−1^)	0.032 ± 0.003
t 1/2	15.5 ± 2.26
AUC 0–t (µgm/mL·h)	164.9 ± 23.2
MRT (h)	11.86 ± 0.69

Cmax: Peak plasma concentration of drug, Tmax: Time at which Cmax was observed, K: elimination rate constant, t 1/2: Elimination half-life, AUC 0–t: Area under the plasma concentration time curve from 0 h to time t, MRT: mean residence time.

**Table 9:** Storage stability of methotrexate nanogel formulations at refrigerated, room, and accelerated temperatures for 3 months.

Storage Conditions			
	Refrigerated	Room	Accelerated
Particle Size (nm)	23.72 ± 5.82	25.81 ± 1.67	32.50 ± 5.78
PDI	0.27 ± 0.41	0.29 ± 0.04	0.36 ± 1.24
Zeta Potential (mV)	−3.38 ± 5.85	−4.78 ± 3.78	−4.99 ± 0.02
pH	6.23 ± 0.91	5.99 ± 0.48	5.03 ± 0.72
Phase Separation	Nil	Nil	Nil
Clarity	+	+	+
Drug Content (%) ± SD	98.86 ± 0.23	98.53 ± 0.88	97.35 ± 2.66
Color Change	++	++	++

Values are expressed as mean ± SD (*n* = 3). + Transparent and clear, ++ Passed.

## Data Availability

All the data available are provided in this manuscript.
